# Neonatal respiratory care in Vietnam: surfactant use and clinical practices in a large neonatal intensive care unit

**DOI:** 10.1186/s12887-025-06409-7

**Published:** 2025-12-22

**Authors:** Hien Vu, Mårten Larsson, Linh Thi Nguyen, Thanh Phuong Thi Tran, Phuong Thu Thi Pham, Toan K. Nguyen, Linus Olson, Anh Duy Nguyen, Thomas Drevhammar, Tobias Alfvén, Nicolas J. Pejovic, Susanna Myrnerts Höök

**Affiliations:** 1 Department of Global Public Health, Karolinska Institutet, Solna, 17177 Sweden; 2 Intensive Care Unit and Poison Control Department, Phu San Hanoi Hospital, Hanoi, Vietnam; 3 Department of Pediatrics, Institution of Clinical Sciences, Sahlgrenska Academy, University of Gothenburg, Gothenburg, 40530 Sweden; 4 Neonatal Department, Phu San Hanoi Hospital, Hanoi, Vietnam; 5 Department of Surgical Gynaecology, Phu San Hanoi Hospital, Hanoi, Vietnam; 6 Department of Women’s and Children’s Health, Karolinska Institutet, Solna, 17177 Sweden; 7 Department of Obstetrics and Gynaecology, Hanoi Medical University, Hanoi, Vietnam; 8 Department of Obstetrics and Gynaecology, Faculty of Medicine, Vietnam National University, Hanoi, Vietnam; 9 Sachs’ Children and Youth Hospital, Stockholm, 11883 Sweden

**Keywords:** Newborn infant, Neonatal intensive care unit (NICU), Respiratory distress syndrome (RDS), Nasal continuous positive airway pressure (nCPAP), Nasal intermittent positive pressure ventilation (NIPPV), Invasive mechanical ventilation (IMV), High-frequency oscillatory ventilation (HFOV), Bronchopulmonary dysplasia (BPD), Neonatal mortality, Surfactant

## Abstract

**Background:**

Neonatal mortality remains a significant health challenge, particularly in low- and middle-income settings, where respiratory distress is a major contributor to mortality. Characterizations of respiratory care practices in the neonatal intensive care unit (NICU), in lower-middle-income contexts in south-east Asia, are notably sparse in the literature. This study aimed to describe the management practices, morbidity, and mortality of newborns requiring respiratory support at a large level III NICU in Hanoi, Vietnam.

**Methods:**

This prospective, descriptive observational study was conducted at Phu San Hanoi Hospital with about 35 000 births in 2023. Infants born alive, requiring respiratory support, and with a birth weight ≥ 700 g were included. Data were collected from September 1 to November 30, 2023, and analysed using descriptive statistics.

**Results:**

During the study period 17% of infants born at the hospital were admitted to the NICU of which n=874, 52% were included in the analysis. The median gestational age was 34.6 weeks, and the median birth weight was 2,150 g. Respiratory distress syndrome (RDS) was the leading cause of respiratory support (41%), followed by transient tachypnoea of the newborn (24%) and early-onset sepsis (13%). Non-invasive respiratory support, especially nasal continuous positive airway pressure (nCPAP) (45%) and nasal intermittent positive airway pressure (NIPPV) (21%), was widely utilized, with NIPPV being more common among larger infants. Invasive mechanical ventilation was required in 23% of infants, with higher rates in lower birth weight groups. The overall mortality rate was 3%, with the highest mortality among extremely low birth weight infants (36%). Surfactant therapy was administered to 37% of infants with RDS which was also the primary indication for surfactant treatment (98%). Of all first-dose surfactant therapies, 92% were given within the first six hours after birth and 49% were administered by the INSURE (intubation-surfactant-extubation) method.

**Conclusions:**

This study provides valuable insights into respiratory care practices and outcomes for infants requiring respiratory support at northern Vietnam’s largest obstetrical hospital. It highlights RDS as a significant contributor to neonatal morbidity and identifies opportunities to enhance non-invasive support and surfactant administration techniques, potentially reducing invasive mechanical ventilation and improving neonatal outcomes.

## Introduction

Globally, neonatal deaths constituted 48% of all 4.8 million under-five deaths in 2023, with the vast majority occurring in low- and middle-income countries. Major causes include preterm birth complications, intrapartum-related events (birth asphyxia) and infections, factors often leading to respiratory distress and necessitating respiratory support in a neonatal intensive care unit (NICU) [1, 2]. 

In recent decades, NICUs in high-resource settings have shifted toward more non-invasive respiratory support due to risks associated with invasive mechanical ventilation (IMV), such as baro- and volutrauma, and eventually bronchopulmonary dysplasia (BPD) [3–9]. The mainstay of treatment is now nasal continuous positive airway pressure (nCPAP) together with nasal intermittent positive pressure ventilation (NIPPV), while IMV is used as a rescue treatment. Simultaneous, less invasive methods for surfactant administration have been adopted to avoid IMV in infants with respiratory distress syndrome (RDS) [10–13]. 

In China, large observational studies over the past decades have also reported a shift from predominantly IMV to non-invasive respiratory support, coinciding with a concurrent reduction in mortality [14, 15]. Neonatal mortality in Vietnam in 2020 was 10 deaths per 1000 live births, falling below the Sustainable Development Goal (SDG) 3.2 target of 12 but showing little further decline in the past decade [2]. Research to improve respiratory care is crucial for further reducing neonatal mortality and morbidity [16]. Properly designed clinical trials rely on observational studies to characterize the clinical landscape, generate hypotheses, determine sample sizes and establish benchmarks.

Recent studies by this research team have described the incidence of intrapartum-related events and delivery room interventions at Phu San Hanoi Hospital (PSH), the largest obstetric hospital in northern Vietnam [17, 18]. Yet, data on the incidence and management of respiratory disorders requiring support in lower-middle-income contexts in south-east Asia remains limited. Understanding such data is essential for designing future interventional studies on neonatal respiratory support in the region.

This study aimed to describe the incidences of diagnoses, respiratory practices, morbidity, and mortality among newborn infants in need of respiratory support admitted to the level III NICU at Phu San Hospital (PSH) in Hanoi, Vietnam, and to detail the use of surfactant therapy for treating RDS.

## Materials and methods

### Study design, setting and population

This descriptive observational study was conducted prospectively at PSH, the largest obstetric facility in northern Vietnam, which recorded 34,458 births and 3,983 preterm infants in 2023. The neonatal department consisted of three units: a NICU (25 beds), a neonatal ward (70 beds), and a Kangaroo Mother Care (KMC) unit (50 beds), supported by 85 nurses and 24 doctors. The nurse-to-patient ratio around the clock was 1:3–6 in the NICU, 1:5–10 in the ward and 1:9–10 in the KMC unit. The neonatal units admitted 5,894 patients in 2023 (mean 491/month), peaking in September (570) and reaching a low in January (426).

According to the local guidelines, nCPAP was the primary mode of non-invasive respiratory support for preterm infants. NIPPV was used as a step-up or step-down from IMV and primary support for term infants with respiratory distress. Supplemental oxygen could be administered via a nasal cannula if no additional pressure support was needed. Both nCPAP and NIPPV were delivered via the RAM-cannula (Neotech; Valencia, CA, US) interface [19]. Conventional IMV was used as first-line invasive mechanical support, and high-frequency oscillatory ventilation (HFOV) was used as a rescue treatment for both preterm and term infants. Surfactant therapy guidelines and indications aligned with the 2022 European Consensus on the Management of RDS [10]. Two types of surfactants were used: poractant alfa (Curosurf^®^, Chiesi Farmaceutici S.p.A, Parma, Italy) at a standard dose of 200 mg/kg and beractant (Survanta^®^, AbbVie Inc. North Chicago, IL, US) at a standard dose of 100 mg/kg. Surfactant therapy is provided to all infants who meet clinical criteria, with costs fully covered by the public health insurance system. For spontaneously breathing infants, surfactant was primarily administered through the INSURE method (intubation–surfactant–extubation), typically without premedication. IMV routinely followed the repeated dosing of surfactant. Full respiratory support with IMV and surfactant therapy was offered from a gestational age (GA) of 26 weeks or a birth weight (BW) of 700 g. Antenatal corticosteroids were given from GA 24 + 0 weeks to GA 36 + 6 weeks, with betamethasone 12 mg in two doses with 24 h between doses. Antibiotics were routinely withdrawn if negative blood culture and no laboratory or clinical signs of sepsis after three days of treatment. Infants requiring surgery, therapeutic hypothermia, extracorporeal membrane oxygenation (ECMO), inhaled nitric oxide, and treatment for multiresistant bacteraemia or congenital metabolic disorders were transferred to the Vietnam National Children’s Hospital (VNCH), located in a neighbouring facility.

Infants were eligible for inclusion if they were born alive at PSH, required any kind of respiratory support (oxygen by nasal cannula, nCPAP, NIPPV, or IMV) and BW ≥ 700 g. There were no exclusion criteria. All infants admitted to the NICU and fulfilling the inclusion criteria from September 1 to November 30, 2023, were included and followed until discharge, transfer or death.

### Data collection

Infants admitted to the NICU during the study period were screened for eligibility via electronic medical records during office hours. Data on demographic and clinical variables were extracted from medical records by two trained resident neonatologists using a standardized case report form with predefined variable definitions. Ambiguities were discussed within the team to ensure consistency across data collectors. Maternal, perinatal and delivery room data were collected from the medical records at inclusion, shortly after admission. Clinical and respiratory care variables were collected from medical records and charts during admission and at discharge. Data variables were entered into the REDCap electronic capture database (Vanderbilt, USA) by non-clinical data officers. The dataset was screened for outliers and missing values and cross-checked with source data retrospectively. A post hoc retrospective chart review was undertaken after completion of the prospective data collection to ascertain transfer indications and clinician-assigned primary causes of death.

### Definitions and diagnostic criteria

All diagnoses in this study were based on the information documented in the medical records by the treating physicians.

- RDS was diagnosed based on respiratory distress after birth in preterm infants and pulmonary X-ray findings in infants at risk (e.g., preterm infants, diabetic pregnancy).
- Transient tachypnoea of the newborn (TTN) was defined as transient respiratory distress present at birth with supportive chest X-ray findings of interstitial fluid and exclusion of other causes.
- Persistent pulmonary hypertension of the newborn (PPHN) was defined as severe hypoxemia accompanied by corresponding cardiac ultrasound findings.
- Meconium aspiration syndrome (MAS) was diagnosed when respiratory distress in an infant born through meconium-stained amniotic fluid with corresponding chest X-ray findings and no other explanatory causes.
- Early-onset sepsis was defined as the onset of symptoms < 72 h after birth and positive blood culture or sustained clinical, radiologic or laboratory findings of infection and pursued antibiotics for at least five days.
- Late-onset sepsis was defined by similar means but with the onset of symptoms ≥ 72 h after birth.
- Pneumothorax was diagnosed by typical findings on a chest X-ray.
- Congenital anomalies were indicated for any malformation affecting organ function or disability (not including persistent ductus arteriosus).
- BPD was defined in the study as a gestational age < 32 weeks and requirement for any kind of respiratory support at a post-menstrual age of 36 weeks; or a gestational age ≥ 32 weeks and requirement for any kind of respiratory support ≥ 28 days.
- Necrotizing enterocolitis (NEC) was diagnosed when clinical, laboratory, and radiographic features were consistent with Bell’s criteria; however, Bell stage was not documented [20].
- All infants < 32 weeks were screened with head ultrasounds within seven days after birth and subsequently within one month of life. Intraventricular haemorrhage (IVH) was diagnosed according to the classification by Volpe, but was not graded in the study CRF; grading was added after a post-hoc retrospective chart review was undertaken [21].

### Sample size

No a priori sample size calculation was performed, as this descriptive observational study included all infants admitted to the NICU at PSH who required respiratory support during the study period. This inclusive approach ensured a comprehensive overview of clinical practice and was necessary for accurate incidence estimates. The data are intended to support hypothesis generation, characterize care patterns, and inform future interventional studies and quality improvement initiatives.

### Statistical analyses

The data were summarized using descriptive statistics. Data analysis was performed via IBM SPSS Statistics, version 29 (IBM Corp., Armonk, NY, USA). Continuous variables are presented as medians with interquartile ranges (IQRs), while categorical variables are expressed as numbers and percentages (%). Kaplan-Meier function in SPSS was used to illustrate the duration of respiratory support. Since the outcomes of transferred patients were unknown, a sensitivity analysis was conducted to assess the potential effect on mortality by modelling two scenarios: (1) all transferred patients were assumed to have died (a conservative ‘worst-case’ scenario), and (2) 50% of transferred patients were assumed to have died (an intermediate hypothetical scenario). These assumptions provide a reasonable range of scenarios to evaluate the robustness of our findings.

We utilized the the AI-based services, Avidnote (Version 2025; Avidemic AB; https://avidnote.com) and ChatGPT Edu (OpenAI) employing their Large Language Models (LLMs)-based editing tool to improve grammar, clarity and readability of the manuscript.

## Results

### Study population and baseline data

During the study period, PSH recorded 9,714 births, resulting in 9,982 newborn infants. Of these, 1,694 (17%) were admitted to the NICU, with 939 requiring respiratory support. As part of data cleaning, 21 duplicate records were identified and removed prior to analysis. Seventeen patients—including nine who were intubated in the delivery room—were transferred to VNCH for higher-level care immediately after birth and were therefore excluded from the analysis. Infants with birth weight below 700 g, for whom respiratory support was limited within a palliative care approach, were also excluded; 26 of 27 in this group did not survive.

Baseline characteristics of the remaining 874 infants (52% of admissions) are presented in Table 1. The median gestational age was 35 weeks and the median birth weight (BW) was 2,250 with females accounting for 42%. Most infants (45%) admitted for respiratory support were low birth weight (LBW, 1500–2499 g) followed by (37%) normal birth weight (NBW, 2500–3999 g); 14% were very low birth weight (VLBW, 1000–1499 g), 4% were extremely low birth weight (ELBW, < 1000 g) and only 1% were high birth weight (HBW, ≥ 4000 g).

**Table 1:** Peripartum and neonatal characteristics according to birth weight of infants receiving respiratory support

	Total	BW, g				
	*n* = 874	ELBW < 1000 *n* = 31	VLBW 1000-1499 *n* = 120	LBW 1500–2499 *n* = 394	NBW 2500-3999 *n* = 320	HBW ≥ 4000 *n* = 9
	**Median (IQR)**					
Gestational age, weeks	34.9 (32.1–37.9)	28.0 (26.1–28.7)	30.0 (29.0–31.4)	34.0 (32.6–35.0)	38.0 (37.0–39.0)	39.0 (38.4–39.6)
Birth weight, g	2250 (1700–2850)	900 (800–900)	1250 (1100–1400)	1950 (1750–2200)	3000 (2800–3300)	4100 (4000–4200)
Apgar 1 min	7 (6–8)	5 (4–6)	6 (6–7)	7 (7–7)	8 (7–8)	8 (8–8)
Apgar 5 min	8 (8–9)	6 (6–7)	7 (7–8)	8 (8–8)	9 (8–9)	9 (9–9)
	**n (%)**					
Female	367 (42.0)	15 (48.4)	54 (45.0)	180 (45.7)	117 (36.6)	1 (11.1)
Caesarian delivery^a^	624 (71.4)	14 (45.2)	70 (58.3)	286 (72.6)	245 (76.6)	9 (100)
Antenatal steroids^b^						
Any dose	365 (41.8)	27 (87.1)	99 (82.5)	225 (57.1)	14 (4.4)	0 (0.0)
Full course	300 (34.3)	23 (74.2)	84 (70.0)	182 (46.2)	11 (3.4)	0 (0.0)
Rupture of membranes > 18h^c^	102 (11.7)	7 (22.6)	21 (17.5)	65 (16.5)	9 (2.8)	0 (0.0)
PPV in delivery room	173 (19.8)	23 (74.2)	60 (50.0)	61 (15.5)	29 (9.1)	0 (0.0)
ETI in delivery room	33 (3.8)	4 (12.9)	9 (7.5)	11 (2.8)	9 (2.8)	0 (0.0)
Chest compressions in delivery room	6 (0.7)	1 (3.2)	2 (1.7)	2 (0.5)	1 (0.3)	0 (0.0)

*Abbreviations*: *BW* Birth Weight, *ELBW* Extremely Low Birth Weight, *VLBW* Very Low Birth Weight, *NBW* Normal Birth Weight, *HBW* High Birth Weight, *PPV* Positive Pressure Ventilation, *ETI* Endotracheal Intubation

Missing data: ^a^*n*=3

^b^*n*=53 and

^c^*n*=12

Caesarean sections constituted 71% of deliveries and were the most common delivery mode for most BW groups, except for ELBW (45%). Antenatal corticosteroids were administered to 87% of the ELBW group, 83% of the VLBW group, and 57% of the LBW group. The median Apgar scores at 1 and 5 min were 7 (IQR 6–8) and 8 (IQR 8–9), respectively, with the lowest scores in ELBW infants and higher scores with increasing BW. Positive pressure ventilation (PPV) in the delivery room was given to 20% of infants, with the highest prevalence observed among those in the ELBW group (74%), compared to just 9% in the NBW group. Across all newborns (174 out of 9,982), the overall incidence of PPV was 1.7%. Endotracheal intubation (ETI) in the delivery room occurred in 4% of cases, primarily among infants with lower birth weights, and accounted for 0.3% (34 out of 9,982) of all newborns.

### Clinical outcomes and treatment

Table 2 summarizes the initial respiratory disorders and support for all infants included in the study. RDS was the leading cause for receiving respiratory support, affecting 41% of admitted infants, with the highest cumulative incidence in lower BW groups. TTN (24%) primarily affected larger infants, while early-onset sepsis was observed in 13% of cases, particularly in the smallest BW groups (VLBW 29% and ELBW 39%). Out of these, nine had positive blood cultures; the remaining were classified as clinical sepsis per the methods description. The cumulative incidences of PPHN and MAS were low, at 2% and 0.5%, respectively.

**Table 2:** Initial respiratory disorder and respiratory support according to birth weight of infants receiving respiratory support

	Total	BW. g				
	*n* = 874	ELBW < 1000 *n* = 31	VLBW 1000-1499 *n* = 120	LBW 1500–2499 *n* = 394	NBW 2500-3999 *n* = 320	HBW ≥ 4000 *n* = 9
**Disorders requiring respiratory support at admission**						
	**n (%)**					
RDS	358 (41.0)	29 (93.5)	112 (93.3)	198 (50.2)	19 (5.9)	0 (0.0)
TTN	213 (24.4)	0 (0)	2 (1.7)	68 (17.3)	136 (42.5)	7 (77.8)
Early onset sepsis^a^	112 (12.8)	12 (38.7)	35 (29.2)	39 (9.9)	25 (7.8)	1 (11.1)
PPHN^b^	21 (2.4)	3 (9.7)	7 (5.8)	6 (1.5)	5 (1.6)	0 (0.0)
MAS	4 (0.5)	0 (0.0)	0 (0.0)	2 (0.5)	2 (0.6)	0 (0.0)
HIE	16 (1.8)	2 (6.5)	4 (3.3)	5 (1.3)	5 (1.6)	0 (0.0)
**Incidence of respiratory support by modality**						
	**n (%)**					
O_2_ nasal cannula	744 (85.1)	17 (54.8)	102 (85.0)	333 (84.5)	283 (88.4)	9 (100.0)
nCPAP	396 (45.3)	28 (90.3)	112 (93.3)	230 (58.4)	26 (8.1)	0 (0.0)
NIPPV^c^	179 (20.5)	14 (45.2)	32 (26.7)	48 (12.2)	83 (25.9)	2 (22.2)
IMV, any mode	197 (22.5)	22 (71.0)	38 (31.7)	72 (18.3)	64 (20.0)	1 (11.1)
Conventional IMV	192 (22.0)	22 (71.0)	37 (30.8)	69 (17.5)	63 (19.7)	1 (11.1)
HFOV	32 (3.7)	10 (32.3)	6 (5.0)	7 (1.8)	8 (2.5)	1 (11.1)
**Duration of respiratory support (** *N* ** = cases with indicated support above)**						
	**Median**,** (IQR)**					
O_2_ nasal cannula, days	1.0 (0.3–2.0)	14.0 (7.0–24.0)	7.0 (3.0–18.0)	1 (0.5–2.0)	0.4 (0.2–1.0)	0.4 (0.2–1.0)
nCPAP, days	1.7 (0.8–4.3)	9.5 (3.0–24.5)	5.0 (2.0–8.0)	1.0 (0.7–2.0)	0.4 (0.2–1.0)	0.0 (0.0)
NIPPV, days	1.0 (0.6–2.0)	4.9 (4.0–9.0)	2.0 (1.0–5.5)	1 (0.5–1.5)	1 (0.3–1.2)	1.7 (0.5–3.0)
Conventional IMV, days	2.0 (1.0–4.0)	2.8 (1.5–6.0)	2.0 (1.0–4.0)	2.0 (1.0–4.0)	1.4 (0.5–2.6)	0.7 (0.7–0.7)
HFOV, days	2.0 (0.8–3.5)	2.5 (0.8–5.0)	1.5 (1.0–2.0)	1.0 (0.8–4.0)	1.5 (0.2–2.0)	3.0 (3.0–3.0)
Total time respiratory support, days	1.5 (0.3–5.0)	29.0 (7.2–50.0)	13.0 (7.0–26.0)	1.8 (0.5–3.5)	0.5 (0.2–1.7)	0.4 (0.2–3.0)

Abbreviations: *BW* Birth Weight, *ELBW* Extremely Low Birth Weight, *VLBW* Very Low Birth Weight, *NBW* Normal Birth Weight, *HBW *High Birth Weight, *RDS* Respiratory distress syndrome, *TTN* Transient tachypnea of the Newborn, *MAS* Meconium aspiration syndrome, *PPHN* Persistent pulmonary hypertension of the newborn, *O*_*2*_ Oxygen, *nCPAP* nasal continuous positive pressure ventilation, *NIPPV* Nasal intermittent positive pressure ventilation, *IMV* Invasive mechanical ventilation, *HFOV* High frequency oscillatory ventilation

Missing data: ^a^*n*=18

^b^*n*=2 and

^c^=1

The overall mortality rate was 3% (*n* = 22), with the ELBW group experiencing the highest rate at 36% (Table 3) Most deaths occurred by the third day, while later mortality was more common among infants with lower birth weights. The main causes for death were respiratory failure in preterm infants, (*n* = 5), septic shock (*n* = 5), congenital anomalies including congenital heart disease (*n* = 5), and pulmonary haemorrhage in preterm infants (*n* = 4); single cases were due to pneumothorax and hydrops, and one was unspecified. A total of 6% of infants were transferred to VNCH for higher-level care during admission, and data up until the transfer were included in the analysis. The proportion of transfers was similar across weight groups, most transfers occurred at the second day of life, while smaller infants tended to be transferred much later in life. The most common indications were congenital heart disease (*n* = 9) and need for gastrointestinal surgery (*n* = 8), followed by congenital diaphragmatic hernia (*n* = 5) and PPHN (*n* = 4); smaller groups included large PDA, exchange transfusion, metabolic disease, and ROP (each *n* = 3), with two cases each of hepatic tumor and congenital anomalies, and single cases of severe thrombocytopenia, hypopharyngeal tumor, CMV pneumonia in an extremely preterm infant, hydrocephalus, hypoxic-ischemic encephalopathy (HIE), seizures, cholestatic jaundice, airway malformation, bronchopulmonary dysplasia (BPD), and one transfer upon family request. The median duration of respiratory support among transferred infants was one day (IQR 0.1–7.1) and 73% (*n* = 38) required IMV treatment. In a sensitivity analysis of mortality outcomes, assuming all transferred patients succumbed, the overall mortality rate increased to 8%. Alternatively, under the assumption that 50% of transferred patients died, the mortality rate rose to 5%.

**Table 3:** Mortality and transfer during admission according to birth weight of infants receiving respiratory support

	Total	BW, g				
	*n* = 874	ELBW < 1000 *n* = 31	VLBW 1000-1499 *n* = 120	LBW 1500–2499 *n* = 394	NBW 2500-3999 *n* = 320	HBW ≥ 4000 *n* = 9
	**n (%)**					
Death during admission	22 (2.5)	11 (35.5)	5 (4.2)	6 (1.5)	0 (0.0)	0 (0.0)
Transfer during admission^a^	52 (5.9)	5 (16.1)	6 (5.0)	22 (5.6)	19 (5.9)	0 (0.0)
	**Median (IQR)**					
Time of death, days	4 (1–7)	6 (1–12)	5 (1–7)	2 (1–3)	0 (0–0)	0 (0–0)
Time of transfer, days	2 (1–8)	68 (63–71)	7.5 (1–24)	2 (1–6)	1 (1–2)	0 (0–0)

Abbreviations: *BW* Birth Weight, *ELBW* Extremely Low Birth Weight, *VLBW* Very Low Birth Weight, *NBW* Normal Birth Weight, *HBW* High Birth Weight

^a^Transfer to higher level of care at Vietnam National Children’s Hospital

**Fig. 1:**
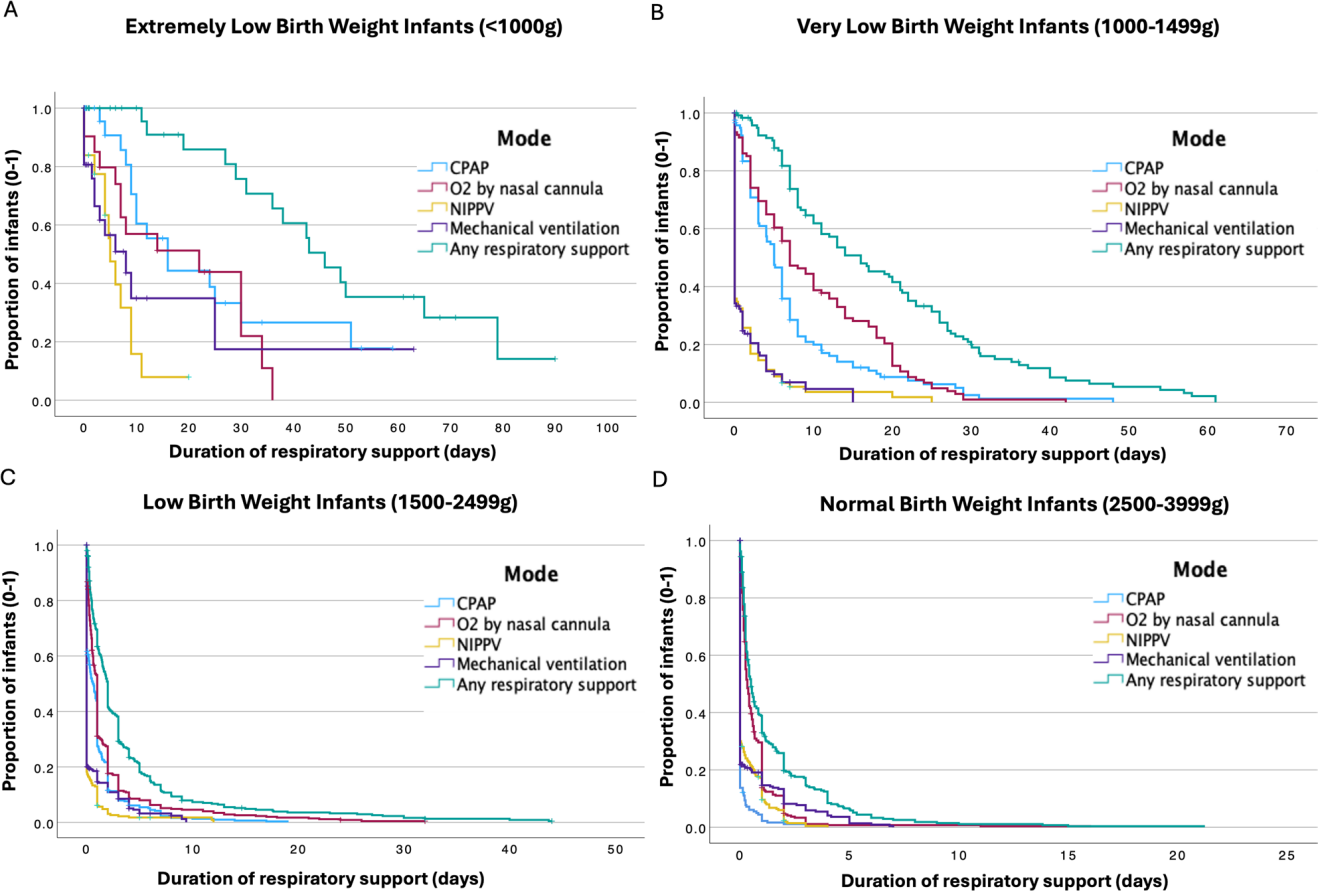
Plots illustrating total number of days (duration) of respiratory support in all infants, across respiratory support modalities, categorized by birth weight (Panels **A**–**D**). Tick marks (+) indicate censored observations (deaths or transfers). **A** Extremely low birth weight infants (*n* = 31, 16 censored). **B** Very Low Birth Weight Infants (*n* = 120, 13 censored) (**C**) Low Birth Weight Infants (*n* = 394, 28 censored). **D** Normal Birth Weight Infants (*n* = 320, 19 censored). High birth weight infants (> 4000 g, *n* = 9) are not shown due to their limited number. *O*_*2*_, Oxygen; *CPAP*, nasal continuous positive pressure ventilation; *NIPPV*, nasal intermittent positive pressure ventilation

Figure 1 illustrates the duration of respiratory support across modalities in all infants, stratified by birth weight category. The curves account for censoring due to death or transfer. Respiratory support was most frequent and prolonged in ELBW infants, with an inverse correlation to birth weight. IMV was typically short-term, while non-invasive support predominated across all groups.

Table 2 also specifies incidence and duration of respiratory support. Supplemental oxygen via nasal cannula was the most common non-invasive modality (85%), with comparable distributions across groups, except for the ELBW group (55%). nCPAP was used in 45% of infants, primarily those with lower BWs, while NIPPV was less common overall (21%) but more prevalent in NBW infants. The duration of non-invasive support was the longest for lower BW groups. IMV was employed in 23% of infants, predominantly conventional IMV (22%), with greater proportion among lower BW groups. HFOV was used in only 4% of instances but also more frequently in lower BW groups.

Clinical outcomes at discharge are detailed in Table 4. A total of 16% of infants in the ELBW group and 4% in the VLBW group met the criteria for BPD. Congenital anomalies were present in 5% of infants and evenly distributed across BW groups. Among infants with anomalies, 13% (6 out of 47) died during admission, and 51% (24 out of 47) were transferred to VNCH, with 75% of the transferred cases considered life-threatening. Heart defects were the most common anomalies (*n* = 27), including 12 cases of ventricular septal defects.

**Table 4:** Clinical outcomes at discharge, according to birth weight of infants receiving respiratory support

	Total	BW, g				
	*n* = 874	ELBW < 1000 *n* = 31	VLBW 1000-1499 *n* = 120	LBW 1500–2499 *n* = 394	NBW 2500-3999 *n* = 320	HBW ≥ 4000 *n* = 9
	**n (%)**					
BPD	14 (1.6)	5 (16.1)	5 (4.2)	4 (1.0)	0 (0.0)	0 (0.0)
NEC^a^	16 (1.8)	4 (12.9)	8 (6.7)	3 (0.8)	1 (0.3)	0 (0.0)
IVH grade I-II^b^	10 (1.1)	5 (16.1)	4 (3.3)	1 (0.3)	0 (0.0)	0 (0.0)
IVH grade III-IV^b^	0 (0.0)	0 (0.0)	0 (0.0)	0 (0.0)	0 (0.0)	0 (0.0)
Pneumothorax	8 (0.9)	0 (0.0)	0 (0.0)	2 (0.5)	6 (1.9)	0 (0.0)
Late onset sepsis^c^	67 (7.7)	18 (58.1)	23 (19.2)	19 (4.8)	6 (1.9)	1 (11.1)
Congenital anomaly^d^	47 (5.4)	1 (3.2)	3 (2.5)	26 (6.6)	17 (5.3)	0 (0.0)
	**Median (IQR)**					
Duration of admission, days	6 (3–18)	58 (7–72)	39 (27–50)	7 (4–16)	3 (2–5)	3 (3–6)

Abbreviations: *BW* Birth Weight, *ELBW* Extremely Low Birth Weight, *VLBW* Very Low Birth Weight, *NBW* Normal Birth Weight, *HBW* High Birth Weight, *BPD* Bronchopulmonary dysplasia, *NEC* Necrotizing enterocolitis, *IVH* Intraventricular haemorrhage

Missing data: ^a^*n*=2

^b^*n*=2

^c^*n*=1

^d^*n*=8

Surfactant therapy was administered to 15% of all infants, with RDS as the primary indication (98%)(Table 5). Among infants diagnosed with RDS, 37% (133 out of 363) received surfactant, predominantly in those with a BW < 1,500 g. In 92% of cases, the first dose was given within 6 h after birth. The INSURE method was used for 49% of first doses, with a median FiO₂ of 40%. The remaining infants were intubated followed by IMV at a median FiO₂ of 50%. Among INSURE cases, 24% required IMV within 72 h of birth. A second surfactant dose was given to 15% of patients on surfactant therapy, all intubated followed by IMV, with most second doses administered between 6 and 24 h after birth.

**Table 5:** Surfactant therapy, according to birth weight of infants in receiving respiratory support

	Total	BW, g				
	*n* = 874	ELBW < 1000 *n* = 31	VLBW 1000-1499 *n* = 120	LBW 1500–2499 *n* = 394	NBW 2500-3999 *n* = 320	HBW ≥ 4000 *n* = 9
	**n (%)**					
Surfactant, any dose	134 (15.3)	18 (58.1)	54 (45.0)	53 (13.5)	8 (2.5)	1 (11.1)
**Indication**						
RDS	131 (97.8)	18 (100.0)	54 (100.0)	53 (100.0)	6 (75.0)	0 (0.0)
MAS	2 (1.5)	0 (0.0)	0 (0.0)	0 (0.0)	2 (25.0)	0 (0.0)
Pneumonia	1 (0.7)	0 (0.0)	0 (0.0)	0 (0.0)	0 (0.0)	1 (100.0)
**Surfactant doses**						
1 dose	114 (85.1)	10 (55.6)	48 (88.9)	47 (88.7)	8 (100.0)	1 (100.0)
2 doses	20 (14.9)	8 (44.4)	6 (11.1)	6 (11.3)	0 (0.0)	0 (0.0)
**1st dose surfactant**						
Age at surfactant^a^						
< 6 h	123 (91.8)	17 (94.4)	51 (94.5)	48 (90.6)	7 (87.5)	0 (0.0)
6–24 h	8 (6.0)	1 (5.6)	0 (0.0)	5 (9.4)	1 (12.5)	1 (100.0)
INSURE	66 (49.3)	7 (38.9)	30 (55.6)	29 (54.7)	0 (0.0)	0 (0.0)
FiO^2^ pre-surfactant, median (IQR)^b^	0.40 (0.35–0.40)	0.40 (0.35–0.40)	0.40 (0.35–0.50)	0.35 (0.30–0.38)	NA	NA
Received IMV < 72 h*	16 (24.2)	2 (28.6)	8 (26.7)	6 (20.7)	0 (0.0)	0 (0.0)
Intubated and IMV	68 (50.7)	11 (61.1)	24 (44.4)	24 (45.3)	8 (100.0)	1 (100.0)
FiO^2^ pre-surfactant, median (IQR)^c^	0.50 (0.35–0.60)	0.50 (0.40–0.70)	0.50 (0.35–0.60)	0.40 (0.35–0.60)	0.65 (0.35–0.80)	1.0 (1.0–1.0)
Alfa poractant (Curosurf)	74 (55.2)	16 (88.9)	42 (77.8)	13 (24.5)	3 (37.5)	0 (0.0)
Beractant (Survanta)	60 (44.8)	2 (11.1)	12 (22.2)	40 (75.5)	5 (62.5)	1 (100.0)
**2nd dose surfactant**						
Age after birth^d^						
< 6 h	1 (5.0)	1 (12.5)	0 (0.0)	0 (0.0)	0 (0.0)	0 (0.0)
6–24 h	13 (65.0)	6 (75.0)	3 (50.0)	4 (66.7)	0 (0.0)	0 (0.0)
24–48 h	5 (25.0)	1 (12.5)	2 (33.3)	2 (33.3)	0 (0.0)	0 (0.0)
Intubated and IMV	20 (100.0)	8 (100.0)	6 (100.0)	6 (100.0)	0 (0.0)	0 (0.0)

Abbreviations: *ELBW* Extremely Low Birth Weight, *VLBW* Very Low Birth Weight, *NBW *Normal Birth Weight, *HBW *High Birth Weight, RDS Respiratory Distress Syndrom, *MAS* Meconium Aspiration Syndrome, *INSURE* Intubation-Surfactant-Extubation, *FiO*^*2*^ Fraction of Inspried Oxygen, *IMV* Invasive Mechanical Ventilation

Missing data: ^a^*n*=3

^b^*n*=1

^c^*n*=3

^d^*n*=1

*Received IMV < 72 h after birth

Caffeine therapy was given to 36% of infants, predominating in the ELBW and VLBW groups (94%), with 45% in the LBW group and none in the NBW and LBW groups.

## Discussion

This prospective observational study offers a comprehensive overview of respiratory care practices, morbidity, and mortality among infants requiring respiratory support in the NICU at the largest obstetrical hospital in northern Vietnam. RDS was the most common diagnosis, affecting approximately 41% of all admitted infants and over 90% of those with birth weights below 1500 g. In response, non-invasive respiratory support—particularly nCPAP and NIPPV—was widely employed, underscoring its central role in current clinical practice. This approach aligns with international evidence supporting the early use of non-invasive ventilation to reduce the need for IMV and its associated complications [22]. The study further highlights the use of surfactant therapy for RDS in Vietnam, with frequent use of INSURE to prevent IMV and low failure rate, which harmonize with international guidelines [10]. These data enhance our understanding of neonatal respiratory care in a high-volume, urban NICU in a south-east Asian context.

### Clinical relevance and comparison with other studies

Few studies have mapped respiratory care practices in the NICU throughout admission for all patients requiring support. However, a recent region-wide multicentre cohort study (*n* = 5584) from Jiangsu province in China by Wang et al. provides an opportunity for comparison with an urban upper-middle income context in East Asia [15]. Additionally, a population-based register study (*n* = 3368), by Norman et al. illustrates respiratory care patterns for very preterm infants in a high-income context in Swedish NICUs [23]. 

In the Chinese cohort. the overall incidence of RDS was 53%. In comparison to our study, the ELBW and VLBW groups had lower rates of RDS, at 79% and 69% respectively. On the other hand, the NBW and HBW groups experienced higher incidences of 31% and 20% respectively. These differences may be due to the varying diagnostic criteria used, with Wang et al. including what they define as secondary RDS, whereas we only consider RDS as caused by surfactant insufficiency in the early phase of life [15]. 

In the Chinese study, at least one dose of antenatal corticosteroids was administered to 47% of ELBW and 51% of VLBW infants, while the rate was 88% for infants with gestational ages < 28 weeks in Sweden [15, 24]. The proportion in the VLBW group in our study is comparable to those observed in Sweden, indicating an active and comprehensive maternal healthcare program at PSH.

The caesarean section rate in our study (71%) was slightly higher than previous reports from this hospital (40–60%), likely due to a selection of high-risk infants requiring respiratory support. It was similar to the Chinese cohort by Wang et al. (66%) but far higher than in Sweden (19%) [15, 25, 26]. Such overuse carries risks for both mother and infant and is strongly associated with neonatal respiratory morbidity, particularly RDS and TTN, which may have contributed to the relatively high incidence of these disorders in our study [27–29]. On the other hand, the notably lower occurrence of MAS in the NBW group (1%) of our study compared to the Wang et al. (5%) is intriguing, and warrants further investigation into potential obstetric factors [15]. 

The overall mortality in our study was 3%, substantially lower than the general mortality in the study by Wang et al. (9%). Potential explanations are differences in inclusion criteria, where the Chinese study population required respiratory support for at least 24 h and our study included any respiratory support without duration threshold. The Chinese study was also a region-wide multicentre study, whereas our research was conducted at a single centre, where some of the most critically ill patients were referred to higher-level care (VNCH) [15]. Our sensitivity analysis suggests that these referrals lowered the overall mortality rate. In Sweden, the one-year mortality for infants born at 30 weeks’ gestation is 2%, whereas it is 9% for those at 26 weeks [30]. 

Our findings revealed that nCPAP was primarily used for infants with birth weights below normal, while NIPPV was more common in NBW infants, aligning with local guidelines; however, evidence for NIPPV’s benefit in term infants remains limited [22]. 

Our study observed that 23% of all infants required IMV, compared to 40% reported in Wang et al.‘s study. When comparing the proportions of infants receiving IMV between the two studies, similarities emerge in groups such as ELBW (71% vs. 72%), VLBW (32% vs. 37%), and LBW (19% vs. 26%). However, more pronounced differences are evident in the NBW group (20% vs. 57%). These discrepancies can, as the mortality rate, probably be partially attributed to the transfer of critically ill term infants to a higher-level care facility (VNCH) in our population, and differences in inclusion criteria [15]. 

Norman et al., revealed that only 9% of infants born at 30 weeks’ gestation, in Sweden, required IMV, approximately three times lower than in our VLBW group. Furthermore, the median duration of IMV in the Swedish cohort was about one day shorter than observed in our study [23, 30]. This indicates potential for improvement in strategies aimed at reducing the need for invasive respiratory support, as reliance on IMV heightens the risk of developing BPD, a chronic lung condition linked to long-term respiratory and neurodevelopmental complications [8, 9, 31]. 

In our study, 37% of infants with RDS and 15% of all infants received surfactant, with use declining by birth weight in parallel with RDS incidence (ELBW 58%, VLBW 45%, LBW 13%, NBW 3%). A similar pattern was seen in a Swedish cohort (< 28 weeks: 79%; 28–31 weeks: 27%; 32–36 weeks: 2%; term: 1%), while Wang et al. reported higher overall use (62% with RDS; 39% of all infants) and a less pronounced decline across birth-weight groups (ELBW 79%, VLBW 70%, LBW 57%, NBW 31%) [15, 32]. The relatively lower use in our setting may reflect the high rate of antenatal corticosteroid administration at PSH—comparable to Sweden—as steroid exposure is strongly associated with reduced surfactant need [33, 34]. Differences in the quality and availability of non-invasive respiratory support may also contribute and could explain the lower surfactant utilization observed in the Swedish cohort [23, 35]. The greater use of surfactant among larger infants in the Wang et al. study may relate to their broader definition of RDS, which included “secondary RDS” (acute respiratory distress syndrome), a classification not applied at PSH or in Sweden [15]. 

The clinical indications for surfactant also varied: in our study, surfactant therapy was almost exclusively administered for RDS, unlike in Sweden, where RDS accounted for 81% of surfactant indications, with MAS the second most common; this pattern underscores the notably low incidence of MAS in our study [32]. Approximately half of surfactant treatments were through the INSURE method, which aligns with international guidelines to minimize IMV requirements [10, 11]. However, the absence of premedication during intubation—a common practice in resource-limited settings—can inflict pain and lead to physiological instability, increased intracranial pressure, and maybe IVH, warranting re-evaluation of practice to enhance infant comfort and outcomes [36]. 

Our study found a lower incidence of BPD compared to Wang et al. (ELBW 16% vs. 24%, VLBW 4% vs. 9%) and in the same range as in Sweden (7% at 28–31 weeks’ gestation) [15, 30]. This discrepancy may be partly due to the transfer of sicker infants to higher-level care before 36 weeks’ postmenstrual age, when BPD is typically diagnosed. Moreover, the palliative care approach for infants with birth weights below 700 g significantly reduces the number of cases at risk for developing BPD at PSH.

The observed delivery room PPV rate of 1.7% is likely underestimated since the analysis included only admitted infants needing respiratory support. This figure surpasses our previous studies’ 0.4–0.6%, possibly due to a more proactive resuscitation approach for extremely preterm infants and differing data collection methods. However, ETI rates in the delivery room remain comparable (0.3% vs. 0.2%) [17, 18]. 

### Study strengths and limitations

The strength of this study lies in its prospective design, which enabled comprehensive data collection on a substantial number of infants within a defined time frame. This approach enhances our understanding of respiratory care management and outcomes within an urban setting in Vietnam, an east-asian, lower-middle-income country, where such data is limited. The amount of missing data was low across variables.

However, several limitations should be noted. As a descriptive observational study, it aims to outline patterns rather than establish risk factors and causal relationships. The three-month study period may limit generalizability due to seasonal or random variations in birth rates and outcomes. While a full year of data would have been preferable, this was not feasible given the burden of manual data collection and the need to provide timely data for subsequent clinical trials. Notably, however, although the study window fell in the hospital’s busiest months, NICU admissions in 2023 were relatively stable (mean 491/month; peak 570 in September, low 426 in January), partially mitigating—though not eliminating—seasonal bias. Conducted at the largest obstetrical centre in northern Vietnam, this single-centre study may limit applicability to other NICUs with varying populations, care practices, or resources. Despite prospective data collection, reliance on medical records raises concerns about documentation inconsistencies, possibly resulting in underreporting of treatments or outcomes. Furthermore, retinopathy of prematurity registration were not performed, limiting a comprehensive assessment of neonatal morbidity.

### Potential areas for improvement and future directions

Our findings emphasize the potential to further reduce reliance on IMV, particularly for infants with BWs < 1500 g, where IMV rates are three times higher than in a high-income setting like Sweden [23]. Enhancing non-invasive support strategies and optimizing surfactant therapy may reduce IMV dependence and improve long-term outcomes. Minimally invasive surfactant techniques, such as administration via supraglottic airway devices (SALSA), help preserve spontaneous breathing and reduce procedural stress, potentially decreasing the need for IMV. A limitation is the absence of preterm-specific devices; nevertheless, clinical trials are underway at PSH to assess feasibility and outcomes (ClinicalTrials.gov: NCT06606444, NCT07098910,) [37]. 

The high rate of caesarean section at PSH reflects a global trend of rising CS rates, most pronounced in East Asia, and poses unnecessary risks to both mothers and infants [27]. A previous study from this hospital highlighted this concern and recommended increasing awareness among pregnant women, strengthening adherence to international guidelines among obstetricians, and fostering organizational support to reverse the trend [25]. These recommendations remain highly relevant and warrant reinforcement.

The notable survival rates for preterm infants at PSH accentuate the high quality of care provided by the institution. However, survival is only one aspect; neurodevelopmental outcomes are equally crucial. A recent study from a NICU in Ho Chi Minh found substantially lower neurodevelopmental outcomes in preterm infants compared to healthy peers [38]. These findings underscore the need for a national neonatal follow-up program, as the impacts of neonatal management may only become apparent years later. In Sweden, although the one-year survival rate at 22–23 weeks’ gestation is around 60%, more than 75% of survivors face neurodevelopmental disorders [24, 39]. 

Overall outcomes from PSH appear comparable to the Chinese cohort, by Wang et al., a positive finding given China’s relatively stronger economy [15, 40]. However, such comparisons are prone to bias due to variations in populations, definitions, and reporting. Since 2012, the Swedish Neonatal Quality Register has provided an excellent tool for benchmarking and improving neonatal care across 37 units in Sweden [41]. The upcoming AsianNeo registry will include networks from nine Asian countries to evaluate outcomes in ill newborns and enhance care quality. Unfortunately, Vietnam is not currently part of this initiative [42]. 

To strengthen neonatal research and clinical care in Vietnam, future efforts should prioritize the development of structured follow-up programs and participation in regional or international registries. These initiatives would enable systematic monitoring of long-term outcomes and provide benchmarks for quality improvement, aligning Vietnam’s neonatal care with regional and global standards.

## Conclusion

This study offers valuable insights into respiratory care practices and outcomes for infants requiring respiratory support at northern Vietnam’s largest obstetrical hospital, highlighting RDS as a significant contributor to neonatal morbidity. It also identifies opportunities to enhance non-invasive support strategies and surfactant administration techniques, potentially reducing IMV reliance. Addressing these areas may lead to improvements in neonatal care and ultimately better health outcomes for vulnerable newborns.

## Data Availability

The datasets used and/or analysed during the current study are available from the corresponding author upon reasonable request.

## Abbreviations

BPD: Bronchopulmonary Dysplasia
CRF: Case Report Form
ECMO: Extracorporeal Membrane Oxygenation
ELBW: Extremely Low Birth Weight
IMV: Invasive Mechanical Ventilation
INSURE: Intubation-Surfactant-Extubation
IVH: Intraventricular Haemorrhage
MAS: Meconium Aspiration Syndrome
NIPPV: Nasal Intermittent Positive Pressure Ventilation
NICU: Neonatal Intensive Care Unit
nCPAP: Nasal Continuous Positive Airway Pressure
NEC: Necrotizing Enterocolitis
PPHN: Persistent Pulmonary Hypertension of the Newborn
RDS: Respiratory Distress Syndrome
SDG: Sustainable Development Goals
TTN: Transient Tachypnea of the Newborn
